# Milk From Cow Fed With High Forage/Concentrate Ratio Diet: Beneficial Effect on Rat Skeletal Muscle Inflammatory State and Oxidative Stress Through Modulation of Mitochondrial Functions and AMPK Activity

**DOI:** 10.3389/fphys.2018.01969

**Published:** 2019-01-17

**Authors:** Giovanna Trinchese, Gina Cavaliere, Eduardo Penna, Chiara De Filippo, Fabiano Cimmino, Angela Catapano, Nadia Musco, Raffaella Tudisco, Pietro Lombardi, Federico Infascelli, Giovanni Messina, Laura Muredda, Sebastiano Banni, Marcellino Monda, Marianna Crispino, Maria Pina Mollica

**Affiliations:** ^1^Department of Biology, University of Naples Federico II, Naples, Italy; ^2^Department of Pharmacy, University of Naples Federico II, Naples, Italy; ^3^Department of Veterinary Medicine and Animal Production, University of Naples Federico II, Naples, Italy; ^4^Department of Clinical and Experimental Medicine, University of Foggia, Foggia, Italy; ^5^Department of Biomedical Sciences, University of Cagliari, Cagliari, Italy; ^6^Department of Experimental Medicine, Section of Human Physiology and Unit of Dietetics and Sports Medicine, University of Campania “Luigi Vanvitelli”, Naples, Italy

**Keywords:** milk, high forage/concentrate ratio, mitochondrial functions, skeletal muscle, oxidative stress, inflammation, AMPK activation

## Abstract

Milk and dairy products are relevant components of daily diet and are part of dietary recommendation in many countries due to their content of key nutrients. However, the relatively high content of saturated fat of the milk and its extensive usage for every age group raises concerns about its potential negative health effects. Therefore, in the last years, several researchers dedicated their attention to milk production and quality. Milk fatty acids profile depend on cow feeding and in particular on the type of forage and concentrate and forage/concentrate ratio. It was demonstrated that feeding dairy cows with a 70/30 forage/concentrate ratio yields milk with a low ω6:ω3 ratio and high CLA levels. In this work, we demonstrated that the supplementation of rats diet with this high forage milk (HFM) results, in the skeletal muscle of these animals, in a reduced lipid content and inflammation levels, and an improved mitochondrial lipid oxidation, and redox status through modulation of AMPK activity.

## Introduction

Skeletal muscle, the largest organ in the body, in most mammals accounts up to ∼45% to 55% body mass and, consequently, is the major determinant of the basal metabolic rate (Zurlo et al., 1990, 1994; Janssen et al., 2000). Since muscle is consuming nearly 80% of insulin-stimulated glucose uptake, it plays a key role in glucose disposal (Thiebaud et al., 1982; Ferrannini et al., 1988). Moreover, it can be recruited to increase energy expenditure several folds in physical activity (Ferrannini et al., 1988; Kelley, 2005; Turner et al., 2014), and interestingly, during prolonged energy demand, muscle has the ability to switch from carbohydrates to fatty acid utilization. In addition, skeletal muscle plays a key role in temperature homeostasis since it can be recruited to produce heat through shivering and non-shivering thermogenesis (Pant et al., 2016; Periasamy et al., 2017). Thus, skeletal muscle is responding to several physiological conditions, adjusting to different intracellular energy state (Kjøbsted et al., 2018). AMPK is working as a key intracellular sensor of energy state, regulating the balance between energy supply and demand (Hardie et al., 2012). In skeletal muscle, AMPK activation has been demonstrated to improve glucose and lipid metabolism (Merrill et al., 1997), and to enhance skeletal muscle oxidative capacity, acting on mitochondrial functions (Winder et al., 2000).

Loss of muscle mass and force is associated with several diseases such as diabetes, cancer, and renal and cardiovascular disease as well as aging-sarcopenia. In all these pathological conditions the mitochondrial functions are greatly influenced, and these modulation of mitochondrial activity affects the reactive oxygen species (ROS) production, that plays a crucial role in muscle function (Porter et al., 2013; Cavaliere et al., 2016; Trinchese et al., 2018). In addition, mitochondria dysfunction, triggering catabolic signaling pathways, leads to muscle atrophy (Sartori et al., 2013, 2014; Romanello and Sandri, 2016). On the other hand, physical activity, improving mitochondrial function, plays beneficial effects in several diseases (Little et al., 2011; Lo Verso et al., 2014).

Mitochondria provide energetic support through the chemiosmotic process of oxidative phosphorylation. Indeed, they oxidize nutrients [glucose, fatty acids (FAs), and some amino acids] and the energy generated by the electron transport is used to phosphorylate ADP to ATP, tightly coupling electron transport and ATP synthesis. However, some of the energy generated by electron transport is uncoupled from ATP synthesis (Stucki, 1980). The degree of mitochondrial uncoupling is affected by the permeability of the mitochondrial inner membrane to hydrogen ions (leak). Indeed, the mitochondrial inner membrane exhibits a basal proton leak pathway that has been estimated to contribute 20–25% to the basal metabolic rate in rats (Rolfe and Brown, 1997). In addition, FAs, natural uncouplers of oxidative phosphorylation, generate a FA-dependent proton leak pathway (Skulachev, 1991). Mitochondrial uncoupling, promoting inefficient metabolism, such as the generation of heat instead of ATP, is a potential treatment for obesity. Therefore, natural molecules or drugs modulating the mitochondrial function and efficiency may be useful in the treatment/prevention of obesity and insulin resistance.

Milk and dairy foods provide abundant amounts of proteins, fats, vitamins and minerals necessary for growth and development (Miller and Jarvis, 2000). Ruminants feeding has an important impact on milk production and quality, and therefore several studies are addressed to this direction, in the attempt to obtain healthier dairy products (Tudisco et al., 2010, 2012, 2014; Wiking et al., 2010). In terms of human health, polyunsaturated fatty acids (mainly ω3) and conjugated linoleic acids (CLA) have beneficial properties, such as anticarcinogenic, antioxidative, and antiatherogenic effects, reduction in the body fat, and stimulation of the immune system (Pariza, 1999). Milk fatty acids profile depend on cow feeding and in particular on the type of forage and concentrate and forage/concentrate ratio (Cruz-Hernandez et al., 2007). Indeed, in previous work it was demonstrated that feeding dairy cows with a 70/30 forage/concentrate ratio yields milk with a low ω6:ω3 ratio and high CLA levels (Cavaliere et al., 2018). The administration of this high forage milk (HFM) to rats resulted in beneficial effects on lipid metabolism, inflammation and oxidative stress in the liver (Cavaliere et al., 2018). Since the skeletal muscle is a chief determinant of resting metabolic rate, we analyzed also in this tissue the effects of HFM on mitochondrial functions and redox state.

## Materials and Methods

### Cow Milk and Chemicals

Chemicals were purchased by Sigma–Aldrich (St. Louis, MO, United States), unless otherwise specified. Milk was purchased from a farm that produces 2 types of commercial milk, low forage milk (LFM) and high forage milk (HFM) from Italian Friesian cows fed 2 different diets with lower or higher F:C ratio, respectively. Chemical composition, ω6/ω3 ratio and CLA content are reported in Table 1 (see Cavaliere et al., 2018 for details).

**Table 1:** Milk chemical composition, ω6, ω3, and CLA.

	LFM	HFM
Protein (%)	3.3 ± 0.2	3.3 ± 0.1
Fat (%)	3.5 ± 0.3	3.7 ± 0.2
Lactose (%)	4.8 ± 0.5	4.7 ± 0.4
ω3	0.317 ± 0.01^b^	1.212 ± 0.09^a^
ω6	2.207 ± 0.08	2.403 ± 0.03
ω6/ω3	6.96 ± 1.00^a^	1.98 ± 0.03^b^
ΣCLA	0.45 ± 0.02^b^	0.79 ± 0.02^a^


*Data are means ± SEM. Along the rows data with different superscripted letters are significantly different (P < 0.05). LFM, low forage milk; HFM, high forage milk.*

### Rat Handling and Feeding

Male Wistar rats (Charles River, Calco, Lecco, Italy) were individually caged in a temperature-controlled room and exposed to a daily 12h–12h light–dark cycle with free access to chow and drinking water. Young animals (60 days old; about 350 g b.w.) were used; one group (*n* = 7) was sacrificed at the beginning of the study to establish baseline measurements. The remaining rats, fed with standard diet, were divided into three experimental groups (*n* = 7 each): two groups were supplemented with equicaloric intake (82 kJ) of LFM or HFM (22 ml/day) for 4 weeks; the group that did not receive milk supplement was used as control. After 4 weeks, the animals were anesthetized by intra-peritoneal injection of chloral hydrate (40 mg/100 g body weight), and blood was taken from the inferior cava. The skeletal muscle was removed and sub-divided; samples not immediately used for mitochondrial preparation were frozen and stored at -80°C.

### Serum and Tissue Parameters

The serum levels of cholesterol and triglycerides were measured with standard procedures. Glucose levels were determined by glucometer (Contour next, Ascensia, Switzerland). The serum levels of insulin (Mercodia AB, Uppsala, Sweden) were measured using commercially available ELISA kits. The serum and tissue levels of tumor necrosis factor-a (TNF-α), interleukin (IL)-1, IL-6, IL-10, monocyte chemoattractant protein 1 (MCP-1; Biovendor R&D, Brno, Czechia), and adiponectin (BBridge International Mountain View, CA, United States) were measured using commercially available ELISA kits.

### Isolation of Mitochondria Skeletal Muscle and Measurements of Mitochondrial Oxidative Capacities and Degree of Coupling

Hind leg muscles were freed of excess connective tissue, finely minced, washed in a medium containing 100 mmol/L KCl, 50 mmol/L TRIS, pH 7.5, 5 mmol/L MgCl_2_, 1 mmol/L EDTA, 5 mmol/L EGTA, 0.1% (w/v) fatty acid-free BSA and treated with protease (3.6 U/g tissue) for 4 min. Tissue fragments were then homogenized with the above medium (1:8 w/v) at 500 rpm (4 strokes/min). Homogenate was centrifuged at 3,000 *g* for 10 min, the resulting supernatant was rapidly discarded, and the pellet was resuspended and centrifuged at 500 *g* for 10 min. The supernatant was then centrifuged at 3,000 *g* for 10 min, and the pellet was washed once and resuspended in suspension medium (250 mmol/L Sucrose, 50 mmol/L Tris, pH 7.5, 0.1% fatty acid-free BSA). In control experiments, we assured the quality of our mitochondrial preparation by checking that contamination of mitochondria by other ATPase-containing membranes was lower than 10%, and addition of cytochrome c (3 nmol/mg protein) only enhanced state 3 respiration by approximately 10%.

Oxygen consumption was measured polarographically with a Clark-type electrode (Yellow Springs Instruments, OH, United States) in a 3-mL glass cell, at a temperature of 30°C. Skeletal muscle mitochondria were incubated in a medium containing 30 mmol/L KCl, 6 mmol/L MgCl_2_, 75 mmol/L sucrose, 1 mmol/L EDTA, 20 mmol/L KH_2_PO_4_ pH 7.0 and 0.1% (w/v) fatty acid-free BSA, pH 7.0. The substrates used were 10 mmol/L succinate + 3.8 μmol/L rotenone, 10 mmol/L glutamate + 2.5 mmol/L malate, 40 μlmol/L palmitoyl-carnitine + 2.5 mmol/L malate or 10 mmol/L pyruvate + 2.5 mmol/L malate. After the addition of 0.3 mmol/L ADP, maximal ADP-stimulated oxygen consumption was measured and taken as state 3, while state 4 was obtained from oxygen consumption measurements at the end of state 3, when ADP becomes limiting. Respiratory control ratio was calculated as state 3/state 4 ratio.

The degree of coupling was determined in skeletal muscle mitochondria as previously reported (Lama et al., 2016) by applying equation 11 by Cairns et al. (1998):

$degree\;ofcoupling=\sqrt{1-{\left(Jo\right)}_{\text{sh}}/{\left(Jo\right)}_{rmunc}},$ where (Jo)sh represents the oxygen consumption rate in the presence of oligomycin that inhibits ATP synthase, and (Jo)unc is the uncoupled rate of oxygen consumption induced by FCCP, which dissipates the transmitochondrial proton gradient. (Jo)sh and (Jo)unc were measured as above using succinate (10 mmol/L) + rotenone (3.75 μmol/L) in the presence of oligomycin (2 μg/mL) or FCCP (1 μmol/L), respectively, both in the absence and in the presence of palmitate at a concentration of 50 μmol/L.

Carnitine palmitoyl-transferase (CPT) system, aconitase and superoxide dismutase (SOD) specific activity were measured spectrophotometrically as previously reported (Flohè and Otting, 1984; Alexson and Nedergaard, 1988; Hausladen and Fridovich, 1996). Catalase (CAT) activity was determined based on the decomposition of H_2_O_2_ at 25°C (Aebi, 1984).

### H_2_O_2_ Measurement

Rate of mitochondrial H_2_O_2_ release was assayed by following the linear increase in fluorescence (ex 312 nm and em 420 nm) due to the oxidation of homovanillic acid in the presence of horseradish peroxidase (Barja, 1998).

### Lipid Content, Redox Status and Detoxifying Enzyme Activities in Skeletal Muscles

Total lipid content in the skeletal muscle was estimated by using the Folch method. Briefly, the skeletal muscle was weighed, chopped into small pieces, thoroughly mixed, and finally homogenized with water (volumes equal to twice the skeletal muscle weight) in a Polytron homogenizer. On appropriate aliquots of the homogenate, lipid content was determined gravimetrically after extraction in chloroform–methanol and evaporation to constant weight by a rotating evaporator (Folch et al., 1957). ATP content in skeletal muscles was measured using commercially available kit (Abcam, ab83355). Reduced (GSH) and oxidized (GSSG) glutathione concentrations in skeletal muscle were measured with the dithionitrobenzoic acid (DTNB)-GSSG reductase recycling assay (Bergamo et al., 2007), the GSH/GSSG ratio was used as an oxidative stress marker. To investigate the detoxifying activity, the enzymatic activities of glutathione S-transferases (GSTs) and quinone-oxidoreductase (NQO1) were evaluated spectrophotometrically in cytoplasmic extracts prepared from rat skeletal muscle (Benson et al., 1980; Habig and Jakoby, 1981).

### ROS Assay

The levels of ROS were determined as previously reported (Montoliu et al., 1994). An appropriate volume of freshly prepared tissue homogenate was diluted in 100 mM potassium phosphate buffer (pH 7.4) and incubated with a final concentration of 5 μM dichloro-fluorescein diacetate (Sigma–Aldrich) in dimethyl sulfoxide for 15 min at 37°C. The dye-loaded samples were centrifuged at 12,500 ×*g* per 10 min at 4°C. The pellet was mixed at ice-cold temperatures in 5 ml of 100 mM potassium phosphate buffer (pH 7.4) and again incubated for 60 min at 37°C. The fluorescence measurements were performed with a HTS-7000 Plus-plate-reader spectrofluorometer (Perkin Elmer, Wellesley, MA, United States) at 488 nm for excitation and 525 nm for emission wavelengths. ROS were quantified from the dichloro-fluorescein standard curve in dimethyl sulfoxide (0–1 mM).

### Lipid Analyses

Aliquots of total lipid extract from tissues were mildly saponified as previously described (Banni et al., 1996) in order to obtain free fatty acids for HPLC analysis. Separation of unsaturated fatty acids was carried out with an Agilent 1100 HPLC system (Agilent, Palo Alto, CA, United States) equipped with a diode array detector as previously reported (Melis et al., 2001). ω3 HUFA score, taken as an index of the impact of the diet on the ratio between ω3 and ω6 highly polyunsaturated fatty acids (HPUFAs), was calculated as the ratio between the sum of the concentrations of ω3 HPUFAs (those with 20 or more carbon atoms and three or more double bonds of ω3 family) and the sum of total ω3 and ω6 HPUFAs: [(20:5ω3 + 22:5ω3 + 22:6ω3)/(20:5ω3 + 22:5ω3 + 22:6ω3 + 20:4ω6 + 20:3ω6 + 22:4ω6 + 22:5ω6)^∗^100] (Stark, 2008).

### Protein Carbonyl and Malondialdehyde Measurements

Protein Carbonyl accumulation (PC), as indicator of protein oxidative modification, was measured in the skeletal muscle as previously reported (Levine et al., 1990).

Malondialdehyde (MDA) levels in the skeletal muscle were determined as indicator of lipid peroxidation (Lu et al., 2009). Tissues were homogenized in 1.15% KCl solution. An aliquot (200 μl) of the homogenate was added to a reaction mixture containing 200 μl of 8.1% SDS, 1.5 ml of 20% acetic acid (pH 3.5), 1.5 ml of 0.8% thiobarbituric acid, and 600 μl of distilled water. Samples were then boiled for 1 h at 95°C and centrifuged at 3000 ×*g* for 10 min. The supernatant absorbance was measured by spectrophotometry at 550 nm and the concentration of MDA was expressed as μmol MDA/mg protein of cell homogenate. A standard curve was prepared using MDA bisdimethylacetal as the source of MDA.

### Western Blot Analysis

Skeletal muscle was homogenized in the lysis buffer (150 mM NaCl, 1 mM EDTA, 1 mM EGTA, 1% Triton X-100, 20 mM Tris–HCl pH 7.5) containing protease and phosphatase inhibitors (Sigma Aldrich). Separation of proteins and western blot analyses were performed as previously described (Chun et al., 2004). Briefly, proteins (25 μg/lane) were separated on 10% SDS–PAGE and transferred to PVDF membranes. Filters were probed with anti-phosphorylated AMPKα antibody (pAMPK, Thr172; Cell Signaling Technology Inc. 1:500) and, after stripping, the same filters were probed with anti-AMPKα antibody (Cell Signaling Technology Inc., Beverly, MA, United States. 1:2000). Secondary antibody, directed against rabbit IgG, was HRP-conjugate (Sigma 1:20000). The signals were detected using Immobilon Western Chemiluminescent HRP Substrate (Sigma).

### Statistical Analysis

All data are presented as means ± SEM. Differences among groups were compared by ANOVA followed by Tukey *post hoc* test to correct for multiple comparisons. Differences were considered statistically significant at *p* < 0.05. All analyses were performed using GraphPad Prism (GraphPad Software, San Diego, CA, United States).

## Results

### Energy Intake

As shown in Table 2, the two milk treatments provided similar gross energy intake, which was significantly higher than control. The LFM and HFM groups received about 86% of energy from chow and 14% from milk.

**Table 2:** Gross energy intake.

	Control	LFM	HFM
Total Energy (kJ)	11180 ± 195^a^	12940 ± 170^b^	13203 ± 178^b^
Chow (kJ)	11180 ± 195	11076 ± 140	11354 ± 132
Milk (kJ)		1864 ± 78	1849 ± 60


*Data are means ± SEM. Along the rows data with different superscripted letters are significantly different (P < 0.05). LFM, low forage milk; HFM, high forage milk.*

### Body Weight, Serum Metabolites and Inflammatory Parameters

**FIGURE 1:**
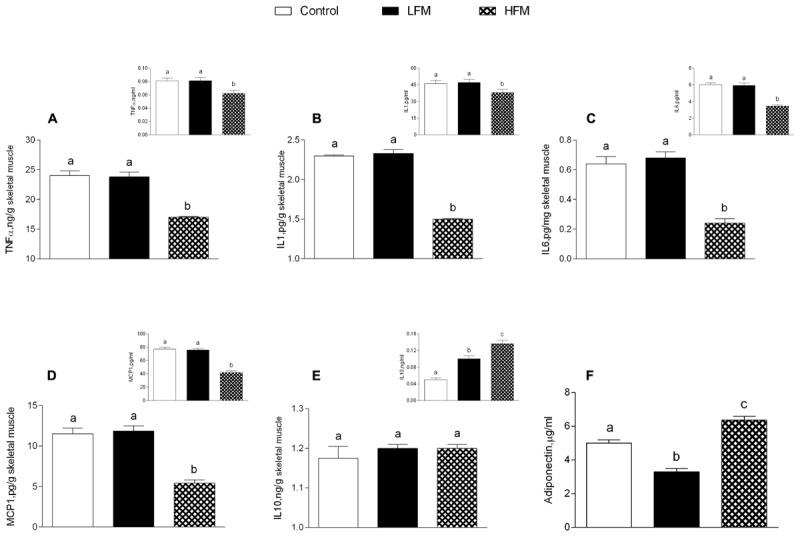
Effects of oral administration of Low Forage Milk (LFM) and High Forage Milk (HFM) on inflammatory parameters. Skeletal muscle levels of TNF-α **(A)**, IL-1 **(B)**, IL-6 **(C)**, MCP1 **(D)**, IL-10 **(E)**, and Adiponectin **(F)**. Insets indicate serum levels of TNF-α **(A)**, IL-1 **(B)**, IL-6 **(C)**, MCP1 **(D)**, and IL-10 **(E)**. Data are presented as means ± SEM from *n* = 7 animals/group. Data with different superscripted letters are significantly different (*p* < 0.05).

Low forage milk- and HFM-treated animals exhibited higher body weight gain compared to control (LFM: 1972 ± 89^a^ kJ, HFM: 1896 ± 68^a^ kJ, control: 868 ± 55^b^ kJ; data with different superscripted letters are significantly different, *P* < 0.05), and higher percentage of body lipid compared to control (LFM: 18,1 ± 1^a^%, HFM: 17,5 ± 0,8^a^%, control: 10,3 ± 0,3^b^%; data with different superscripted letters are significantly different, *P* < 0.05). Glucose, insulin, triglycerides, cholesterol serum levels and HOMA index were not significantly different in the three groups of animals (data not shown) indicating that these parameters are not affected by LFM or HFM administration, in agreement with previously reported data (Cavaliere et al., 2018). Interestingly, tumor necrosis factor-α (TNF-α), IL-1, IL-6, and MCP1 levels significantly decreased in serum and skeletal muscle of HFM-fed animals compared to controls and LFM-fed rats (Figures 1A–D), indicating a possible anti-inflammatory role of HFM. This hypothesis has been further supported by the fact that IL-10, an anti-inflammatory cytokine, was found to increase in the serum of LFM group and even more in the HFM group compared to controls (Figure 1E inset), although no difference was found in skeletal muscle (Figure 1E). Adiponectin levels significantly decreased in LFM, and significantly increased in HFM group compared to the other two groups (Figure 1F).

### Mitochondrial Function and Efficiency

**FIGURE 2:**
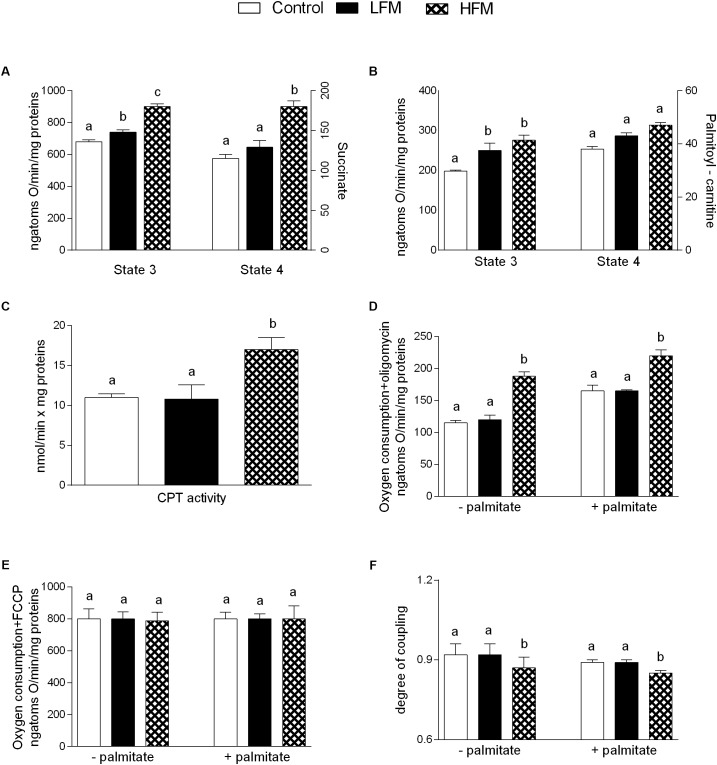
Effects of oral administration of Low Forage Milk (LFM) and High Forage Milk (HFM) on skeletal muscle mitochondrial parameters. Skeletal muscle mitochondrial respiration rates measured in the presence of succinate **(A)** or palmitoyl-carnitine **(B)** as substrates, carnitine-palmitoyl transferase (CPT) activity **(C)**, oxygen consumption in the presence of oligomycin **(D)**, or uncoupled by FCCP **(E)**, degree of coupling values calculated from oxygen consumption in the presence of oligomycin and uncoupled by FCCP **(F)**. Data are presented as means ± SEM from *n* = 7 animals/group. Data with different superscripted letters are significantly different (*p* < 0.05).

Mitochondrial state 3 respiration, evaluated using succinate or palmitoyl-carnitine as substrates (to detect fatty acid oxidation), was increased in LFM- and HFM-fed animals in comparison with the control (Figures 2A,B). Mitochondrial state 4 respiration, using succinate as substrates was increased in HFM-fed animals in comparison with the other two groups (Figure 2A), while no change was observed using palmitoyl-carnitine as substrates (Figure 2B). The high-quality of mitochondrial preparations was tested by evaluation of RCR values (data not shown). CPT activity was increased in HFM-fed animals compared with the other two groups (Figure 2C).

Oligomycin state 4 respiration was significantly higher, both in the absence and in the presence of palmitate, in HFM-fed animals compared to LFM and control rats (Figure 2D), while no variation was found in maximal FCCP-stimulated respiration (Figure 2E). As a consequence, skeletal muscle mitochondrial energetic efficiency, assessed as degree of coupling, was significantly lower in HFM-fed animals compared with the other two groups, both in the absence and in the presence of palmitate (Figure 2F). We also tested the ATP level in skeletal muscle and we did not observe any difference among the three groups of animals (Control 1392 ± 57, LFM: 1495 ± 33, HFM: 1444 ± 64 pmol ATP/mg tissue).

### Oxidative Stress and Antioxidant Activity

**FIGURE 3:**
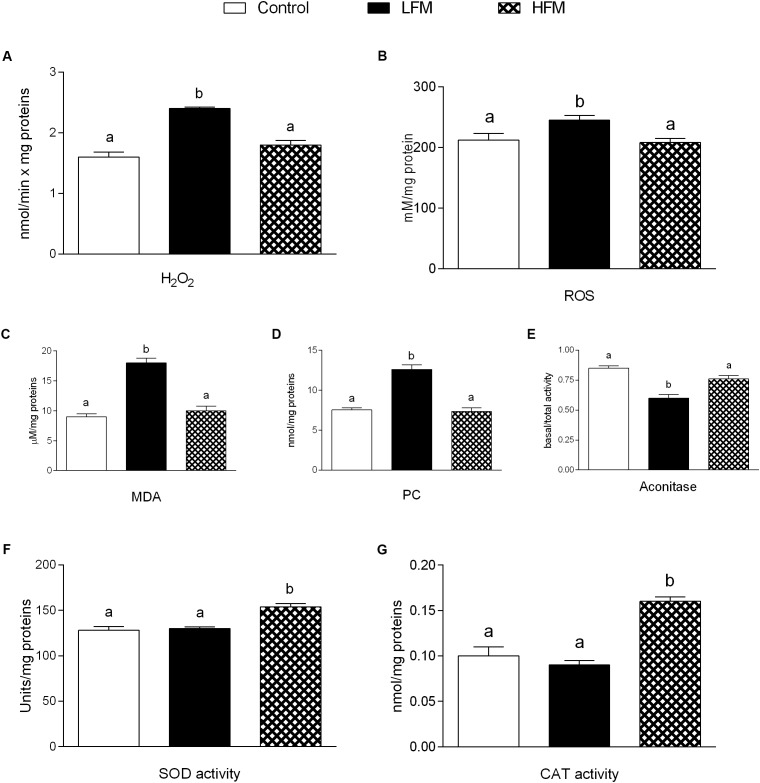
Effects of oral administration of Low Forage Milk (LFM) and High Forage Milk (HFM) on oxidative stress and antioxidant activity. H_2_O_2_ yield **(A)**, ROS **(B)**, malondialdehyde (MDA, **C**), Protein Carbonyl (PC, **D**) levels, basal/total aconitase ratio **(E)**, superoxide dismutase (SOD, **F**) and Catalase (CAT, **G**) activity. Data are presented as means ± SEM from *n* = 7 animals/group. Data with different superscripted letters are significantly different (*p* < 0.05).

H_2_O_2_ yield significantly increased in mitochondria of LFM-fed animals compared with the other two groups (Figure 3A). ROS, MDA and PC in skeletal muscle of LFM-fed animals was significantly higher than in the other groups (Figures 3B–D). Accordingly, the ratio basal/total aconitase activity, as intramitochondrial sensor of redox status, significantly decreased in LFM-fed animals (Figure 3E). In line with this, the antioxidant SOD and CAT activity significantly increased in HFM-fed animals compared with the other two groups (Figures 3F,G).

### Detoxifying Defenses

**FIGURE 4:**
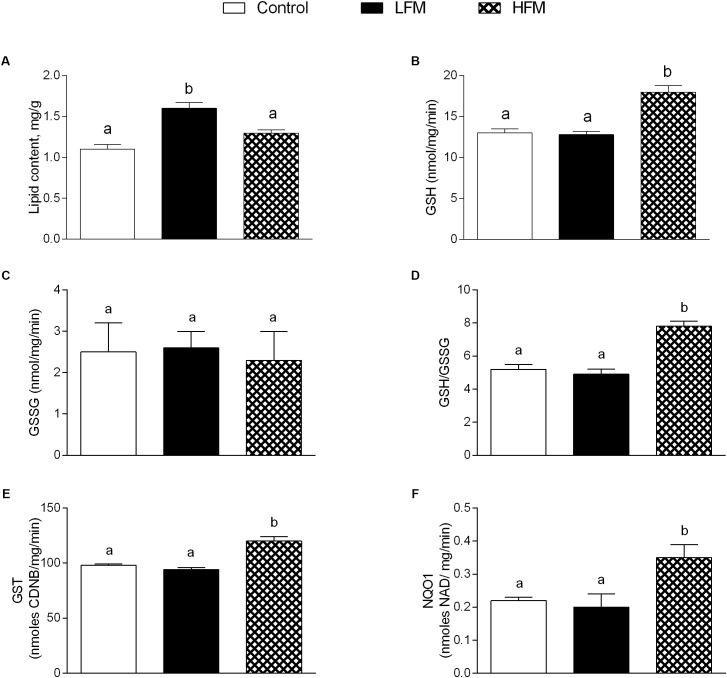
Effects of oral administration of Low Forage Milk (LFM) and High Forage Milk (HFM) on lipid content and detoxifying defenses in skeletal muscle. Skeletal muscle total lipid content **(A)**, GSH content **(B)**, GSSG content **(C)**, GSH/GSSG ratio **(D)**, GST activity **(E)**, and NQO1 activity **(F)**. Data are presented as means ± SEM from *n* = 7 animals/group. Data with different superscripted letters are significantly different (*p* < 0.05).

The lipid content in skeletal muscle of LFM-fed animals was significantly higher than in the other groups (Figure 4A). HFM treatment improved the antioxidant state and cytoprotective enzyme activities. Indeed, while GSH levels significantly increased in HFM-fed animals, compared to the other groups (Figure 4B), GSSG content didn’t change (Figure 4C), resulting in an increased GSH/GSSG ratio in the HFM animal (Figure 4D). In addition, the activities of GST and NQO1 were significantly higher in HFM-fed animals compared with the other two groups (Figures 4E,F).

### Fatty Acid Analysis

**FIGURE 5:**
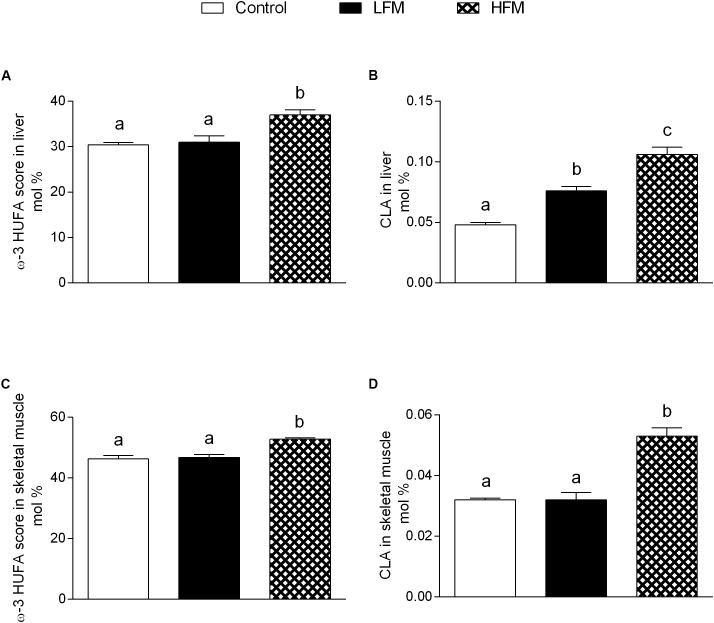
Effects of oral administration of Low Forage Milk (LFM) and High Forage Milk (HFM) on lipid content in liver and skeletal muscle. ω3 HUFA score in liver **(A)** and skeletal muscle **(C)**, and CLA content in liver **(B)**, and skeletal muscle **(D)**. Data are presented as means ± SEM from *n* = 7 animals/group. HUFA, highly unsaturated fatty acid; CLA, conjugate linoleic acid. Data with different superscripted letters are significantly different (*p* < 0.05).

High forage milk-treated animals exhibit significantly increased ω3 HUFA score in liver (Figure 5A) and skeletal muscle (Figure 5C) compared to control and LFM-treated animals. CLA significantly increased in liver of LFM- and HFM-treated animals compared to control (Figure 5B), and significantly increased in skeletal muscle of HFM-treated animals compared to the other two groups (Figure 5D).

### Activation of AMPK

**FIGURE 6:**
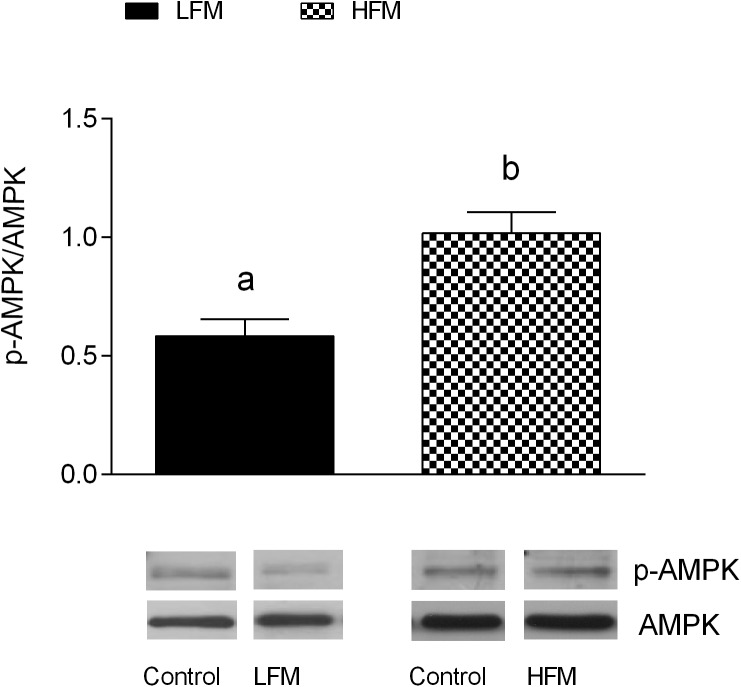
Effects of oral administration of Low Forage Milk (LFM) and High Forage Milk (HFM) on AMPK activation. Data are presented as ratio between milk-treated group and control group (treated/control). Level of AMPK phosphorylation was measured by Western blot as intensity of AMPK phosphorylated at Thr172 (p-AMPK) normalized with total AMPK in the same samples (means ± SEM, *n* = 5). Insets: representative immunoreactive signals for pAMPK, and AMPK in control, LFM and HFM groups. Data with different superscripted letters are significantly different (*p* < 0.05).

To elucidate the possible molecular pathway underlying the beneficial effect of milk supplementation, we analyzed the activity level of AMPK in the three groups of animals. As shown in Figure 6, in HFM group we found an enhanced AMPK phosphorylation levels, compared to LFM.

## Discussion

Our data demonstrated, for the first time, that the administration of HFM in rat model system, reduces lipid content and inflammation in skeletal muscle, and improves redox state modulating mitochondrial functions and increasing detoxifying enzymes activity. These protective effects of HFM may be mediated, at least in part, by activation of skeletal muscle AMPK. These results are particularly relevant in view of the crucial role played by skeletal muscle in metabolic homeostasis of the whole body.

A low grade of inflammation is associated with chronic metabolic disease, such as obesity, in both human and animals. Therefore, in our experiments, we analyzed the influence of HFM in inflammatory state. We show that HFM administration decreases TNFα, IL-1β, IL-6 and MCP1 in both serum and skeletal muscle, and increases IL-10, an anti-inflammatory interleukin, in the serum. In addition, our data provided the first indication that the anti-inflammatory effect of HFM may be, at least partially, mediated by activation of AMPK, the downstream component of a serine/threonine protein kinase cascade involved in the regulation of cellular energy homeostasis. Indeed, AMPK, recognized as an anti-inflammatory protein (Salt and Palmer, 2012), was found to inhibit the synthesis of proinflammatory cytokines and promote the expression of IL-10 (Galic et al., 2011). The decreased inflammation is associated to a decrease in oxidative stress (Spagnuolo et al., 2015; Cavaliere et al., 2016). Since the main sites of ROS production are mitochondria, we analyzed the modulation of skeletal muscle mitochondrial function and efficiency following the administration of LFM and HFM. We observed that LFM and even more HFM-fed rats exhibited increased respiratory capacity and increased fatty acid oxidation. In HFM-fed rats, this increase in fatty acid oxidation is associated to an enhancement of CPT activity, which would further increase entry of long-chain FFAs into mitochondria. Thus, the consequent increase in lipid oxidation explains the decreased load of skeletal muscle lipid content found in these rats. The enhancement of CPT activity may be modulated by AMPK. Indeed, the activation of AMPK decreases the expression of lipogenic genes and increases the phosphorylation of acetyl-CoA carboxylase (ACC), leading to a reduction in malonyl-CoA, which regulates fatty acid oxidation through the inhibition of CPT (Gaidhu et al., 2009). Moreover, AMPK signaling pathway is the main target of adiponectin in promoting fatty acid oxidation (Hernandez-Aguilera et al., 2013). Therefore, the activation of AMPK in HFM group is consistent with the increased level of adiponectin that we observed in the same group.

In HFM group was also observed a reduced mitochondrial energy efficiency that may contribute to fat burning. Indeed, in these conditions, more substrates are burned to produce the same amount of ATP, creating a thermogenic effect. Taken together, the increase in fatty acid oxidation and the decrease in mitochondrial energy efficiency may explain the decrease in lipid content and MDA levels observed in skeletal muscle of HFM-fed animals. The reduced levels of MDA, index of lipid peroxidation, is particularly relevant since the lipid peroxidation is the first step on the way to the development of insulin resistance and its associated diseases. The improvement in mitochondrial functions found in HFM-fed rats could be associated to the increase in CLA levels and ω-3 HUFA score detected in skeletal muscle of these animals. Indeed, it is well-accepted that CLA and ω-3PUFA positively affect mitochondrial activity (Mollica et al., 2014; Cavaliere et al., 2016). Other positive metabolic effects of CLA are related to its ability to increase ω-3 HUFA score in the liver of obese rats with concomitant decrease of fat deposition (Piras et al., 2015), and to increase plasma n-3 HUFA score in hypercholesterolemic subjects (Pintus et al., 2013).

The beneficial effects of HFM administration are indicated also by the increased activities of detoxifying enzymes (GST-NQO1), and by reduction of oxidative stress, indicated by a decrease in H_2_O_2_ production, ROS and CP levels, and an increase in aconitase, SOD and CAT activity. Accordingly, it was recently demonstrated that mitochondrial SOD upregulation leads to the activation of AMPK (Hart et al., 2015). The decreased ROS production may be associated to a decrease in mitochondrial efficiency, since the mitochondrial uncoupling plays a natural antioxidant effect, keeping the mitochondrial membrane potential below the critical threshold for ROS production (Skulachev, 1991; Mailloux and Harper, 2011).

From another point of view, it is important to consider that increased ROS production and reduced antioxidant capacity may also be responsible of altered vascular tone (Gliemann et al., 2016). Regulation of vascular tone is, at least in part, mediated by AMPK through several mechanisms, as stimulation of nitric oxide release in endothelial cells (García-Prieto et al., 2015). Therefore, the correct control of AMPK activation may play a key role in providing an appropriate blood flow to the muscle to enhance the protection from pro-oxidant stimuli.

## Conclusion

In conclusion, our study highlights that rat dietary supplementation with HFM decreases inflammatory status and improves lipid oxidation through AMPK activation in skeletal muscle. Since mitochondrial metabolism and regulation are similar in skeletal and cardiac muscle, that are both striated, our data raise the possibility that consumption of HFM may have beneficial effects also on cardiovascular functions, counterbalancing the oxidants content of western diet. This hypothesis requires dedicated further investigations.

In addition, the beneficial effects elicited by HFM may correlate to its ability to improve redox status and mitochondrial functions possibly mediated by its enriched contents of CLA and ω-3 fatty acids. Further studies are necessary to precisely identify the molecules responsible for the beneficial effects of HFM.

## Ethics Statement

This study was carried out in strict accordance with the Institutional Guidelines and complied with the Italian D.L. no. 116 of January 27, 1992 of Ministero della Salute and associated guidelines in the European Communities Council Directive of November 24, 1986 (86/609/ECC). All animal procedures reported herein were approved by the Institutional Animal Care and Use Committee (CSV) of University of Naples Federico II.

## Author Contributions

MPM and MC conceived and designed the experiments, analyzed the data, and wrote the manuscript. GT, GC, EP, CDF, FC, AC, NM, and LM performed the experiments, collected the data, and performed the data analyses. RT, PL, FI, GM, SB, and MM contributed to the discussion and to the editing of the manuscript. MPM was the guarantor of this work and, as such, had full access to all the data in the study and takes responsibility for the integrity of the data and the accuracy of the data analysis.

## Conflict of Interest Statement

The authors declare that the research was conducted in the absence of any commercial or financial relationships that could be construed as a potential conflict of interest.
